# Prolonged treatment of genetically obese mice with conjugated linoleic acid improves glucose tolerance and lowers plasma insulin concentration: possible involvement of PPAR activation

**DOI:** 10.1186/1476-511X-4-3

**Published:** 2005-01-10

**Authors:** Ed Wargent, Matthew V Sennitt, Claire Stocker, Andrew E Mayes, Louise Brown, Jacqueline O'Dowd, Steven Wang, Alexandra WC Einerhand, Inge Mohede, Jonathan RS Arch, Michael A Cawthorne

**Affiliations:** 1 Clore Laboratory for Life Sciences, University of Buckingham, Buckingham, MK18 1EG, UK; 2 Biosciences division, Unilever Research, Colworth Laboratory, Sharnbrook, Bedfordshire, MK44 1LQ, UK; 3 Lipid Nutrition, Loders Croklaan BV, PO Box 4, 1520 AA Wormerveer, The Netherlands

## Abstract

**Background:**

Studies in rodents and some studies in humans have shown that conjugated linoleic acid (CLA), especially its *trans*-10, *cis*-12 isomer, reduces body fat content. However, some but not all studies in mice and humans (though none in rats) have found that CLA promotes insulin resistance. The molecular mechanisms responsible for these effects are unclear, and there are conflicting reports on the effects of CLA on peroxisomal proliferator-activated receptor-γ (PPARγ) activation and expression. We have conducted three experiments with CLA in obese mice over three weeks, and one over eleven weeks. We have also investigated the effects of CLA isomers in PPARγ and PPARα reporter gene assays.

**Results:**

Inclusion of CLA or CLA enriched with its *trans*-10, *cis*-12 isomer in the diet of female genetically obese (*lep^ob^*/*lep^ob^*) mice for up to eleven weeks reduced body weight gain and white fat pad weight. After two weeks, in contrast to beneficial effects obtained with the PPARγ agonist rosiglitazone, CLA or CLA enriched with its *trans*-10, *cis*-12 isomer raised fasting blood glucose and plasma insulin concentrations, and exacerbated glucose tolerance. After 10 weeks, however, CLA had beneficial effects on glucose and insulin concentrations. At this time, CLA had no effect on the plasma TNFα concentration, but it markedly reduced the plasma adiponectin concentration. CLA and CLA enriched with either isomer raised the plasma triglyceride concentration during the first three weeks, but not subsequently. CLA enriched with its *trans*-10, *cis*-12 isomer, but not with its *cis*-9, *trans*-11 isomer, stimulated PPARγ-mediated reporter gene activity; both isomers stimulated PPARα-mediated reporter gene activity.

**Conclusions:**

CLA initially decreased but subsequently increased insulin sensitivity in *lep^ob^*/*lep^ob ^*mice. Activation of both PPARγ and PPARα may contribute to the improvement in insulin sensitivity. In the short term, however, another mechanism, activated primarily by *trans*-10, *cis*-12-CLA, which probably leads to reduced adipocyte number and consequently reduced plasma adiponectin concentration, may decrease insulin sensitivity.

## Background

The term conjugated linoleic acid (CLA) refers to a mixture of positional and geometric isomers of linoleic acid (*cis*-9, *cis*-12-octadienoic acid). The major components of CLA, are the *cis*-9, *trans*-11 (c9, t11) and the *trans*-10, *cis*-12 (t10, c12) isomers, both of which have biological activities. The t10, c12-isomer is the one primarily responsible for the effects of CLA on weight gain and insulin sensitivity. The CLA used in the present study contains these isomers in roughly equal proportions.

CLA may be of benefit in cancer, atherosclerosis and possibly some disorders of the immune system. In addition, it reduces weight gain and fat accretion in rats and mice. Some studies have found that CLA causes fat loss in humans [1-4], and two studies have shown weight loss[2,5], but another did not find any significant effect of CLA on body composition or body weight [6]. More marked effects of CLA on body weight and body composition have been obtained in rats and especially mice, possibly because the rate of energy expenditure relative to energy stores is much higher in rodents than in humans.

In previous studies, CLA or t10, c12-CLA has exacerbated glucose tolerance and raised plasma insulin levels in normal and genetically obese (*lep^ob^*/*lep^ob^*) mice, despite causing weight loss [7-10]. Both t10, c12-CLA and c9, t11-CLA have also been reported to cause insulin resistance in humans [11-13], although in other studies neither CLA nor its major isomers affected insulin resistance significantly [14-17]. By contrast, CLA or t10, c12-CLA improved glucose tolerance in Zucker fatty *lep^fa^*/*lep^fa ^*and Zucker diabetic fatty *lep^fa^*/*lep^fa ^*rats [18-21]. One difference between the rodent species is that peroxisomal proliferator-activated receptor (PPAR)α-regulated genes are more responsive to CLA in mice than in rats [22], but this would be expected to correlate with improved insulin sensitivity in mice rather than rats [23].

By contrast with this result for PPARα knockout mice, at least one response to CLA depends on expression of PPARγ: the beneficial effect of CLA in a mouse model of colitis was absent in mice that lacked PPARγ in the colon [24]. In view of the variety of reports of CLA's effects on PPARγ activity and expression, it is possible that PPARγ responses may vary between species; the different proportions of isomers in the CLA used in different studies may also be important [25]. Some reports describe activation of PPARγ by CLA or t10, c12-CLA [18,33]; others describe little or no agonist activity [10], but antagonism of rosiglitazone [25,27]. One report describes increased expression of PPARγ mRNA in white adipose tissue of rats [28] and another describes increased expression in liver of mice [10], but studies in isolated adipocytes or of adipose tissue from treated mice show decreased expression [25,27,29,30].

Since PPARγ agonists increase insulin sensitivity but promote adipogenesis, decreased activity of PPARγ in adipose tissue could explain why CLA reduces obesity but increases insulin resistance. Various other mechanisms have also been suggested to explain why CLA exacerbates insulin sensitivity despite causing loss of fat. One of these is increased expression of tumour necrosis factor (TNF)α, since TNFα is associated with apoptosis of adipocytes but causes insulin resistance. TNFα mRNA levels were markedly increased in isolated adipocytes from normal mice that had been fed on CLA for as little as four days [8]. Surprisingly however, serum TNFα levels were reduced by CLA in both normal rats [31] and mice [32]. Moreover CLA reduced the expression of TNFα in mouse macrophages [33].

Although most studies in mice have found that CLA exacerbates glucose tolerance and raises the plasma insulin concentration, in one study treatment of *lep^db^*/*lep^db ^*mice with CLA for eleven weeks improved glucose tolerance and reduced plasma insulin concentration during the glucose tolerance test, indicating improved insulin sensitivity. Treatment for five weeks also improved glucose tolerance but plasma insulin was raised [34]. It is sometimes difficult to interpret studies in *lep^db^*/*lep^db ^*mice because β-cell failure as well as insulin resistance affects glucose homeostasis, and glycosuria can cause weight loss. Therefore, to investigate the effect of CLA on glucose homeostasis further, we have conducted four experiments in *lep^ob^*/*lep^ob ^*mice. We have included rosiglitazone as a comparator in one experiment and compared the effects of t10, c12- and c9, t11-enriched CLA with CLA in another experiment. The first three experiments were over 22 days, whereas the fourth was extended to eleven weeks and included measurements of plasma TNFα and adiponectin. An intriguing finding is that whereas CLA initially exacerbated glucose tolerance and raised the plasma insulin level, after ten weeks it began to improve glucose tolerance and lower the plasma insulin levels.

We also describe activation of PPARγ by t10, c12 but not by c9, t11-CLA, whereas PPARα was activated by both isomers.

## Results

### Weight gain

**Figure 1:**
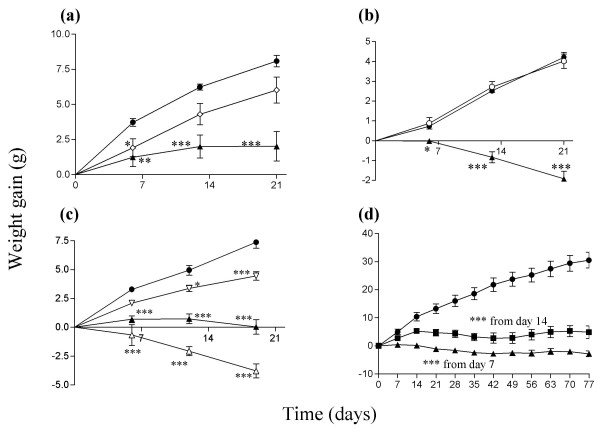
Weight gain in mice fed on diets that contained (doses per kg diet) (a) experiment 1: sunflower oil (●, 15 g), rosiglitazone (◇,10 mg), CLA (▲, 15 g); (b) experiment 2: chow only (○), sunflower oil (●, 25 g), CLA (▲, 25 g); (c) experiment 3: sunflower oil (●, 25 g), CLA (▲, 25 g), t10, c12-CLA (△, 25 g), c9, t11-CLA (▽, 25 g); (d) experiment 4: sunflower oil (●, 25 g), CLA (10 g) plus sunflower oil (15 g) (■), CLA (▲, 25 g). *P < 0.05; **P < 0.01: ***P < 0.001 compared to sunflower oil group.

Inclusion of CLA in the diet of genetically obese (*lep^ob^*/*lep^ob^*) mice reduced their body weight, or weight gain compared to mice fed on a diet containing a similar amount of sunflower oil (Figure 1). Mice fed on chow supplemented with sunflower oil gained weight at the same rate as mice fed on chow alone (Figure 1b). The effect of CLA on weight gain appears to be primarily due to t10, c12-CLA, since CLA enriched to 90% with this isomer had more effect on weight gain than CLA containing 50% t10, c12-CLA and 50% c9, t11-CLA (Figure 1c). CLA that was enriched to 90% with the c9, t11 isomer had only a small effect on weight gain, and this may have been due to the 10% t10, c12-CLA. Food intake was measured over two days in experiment 4 and was not reduced by CLA (food intake per group: control, 33.1 and 37.5 g; CLA 10 g/kg diet, 35.1, 43.4 g; CLA 25 g/kg diet, 39.9, 39.6). Rosiglitazone (10 mg/kg diet) reduced body weight (Figure 1a) but to a lesser extent than CLA (15 g/kg diet).

### Tissue weights and body length

The weight of the parametrial white fat pads was reduced by CLA and by CLA enriched with t10, c12- but not c9, t11-CLA (Tables 1 and 2). The weight of the interscapular brown fat pad was reduced by CLA in experiment 4 (Table 2) but not in experiment 3 (Table 1). This may be because experiment 4 lasted for 11 weeks, whereas experiment 3 lasted for only 3 weeks and the control brown fat pad was more than three times heavier at the end of experiment 4 than at the end of experiment 3, presumably due primarily to its lipid content.

**Table 1:** Terminal tissue weights and NEFA levels in experiment 3. NEFA levels were measured in 5h-fasted mice on day 13. Termination was on day 22. Supplements were included in the diet at concentrations of 25 g/kg.

	Sunflower oil	CLA	t10, c12-CLA	c9, t11-CLA
White adipose wt (g)	0.95 ± 0.12	0.49 ± 0.05^+^	0.39 ± 0.06^+^	0.82 ± 0.04
Brown adipose wt (g)	0.21 ± 0.03	0.23 ± 0.02	0.27 ± 0.11	0.18 ± 0.03
Liver wt (g)	2.02 ± 0.15	3.17 ± 0.05^+^	3.25 ± 0.10^+^	2.76 ± 0.18^+^
Pancreas wt (g)	0.12 ± 0.02	0.12 ± 0.01	0.11 ± 0.02	0.20 ± 0.02
NEFA (mM)	1.09 ± 0.09	1.13 ± 0.14	0.90 ± 0.07	0.82 ± 0.11

+ P < 0.001; n = 6

**Table 2:** Terminal tissue weights and plasma hormones and NEFA levels in experiment 4. NEFA levels were determined in 5h-fasted mice on the days shown. Other measurements were in fed mice on day 77.

	Sunflower oil	1% Clarinol A80	2.5% Clarinol A80
Body length (mm)	95.0 ± 1.0	85.7 ± 0.3^+^	86.4 ± 1.4^+^
White adipose wt (g)	2.11 ± 0.15	0.63 ± 0.07^+^	0.19 ± 0.02^+^
Brown adipose wt (g)	0.74 ± 0.07	0.33 ± 0.04^+^	0.007 ± 0.005^+^
Liver wt (g)	4.96 ± 0.17	6.27 ± 0.58	6.77 ± 0.36*
Pancreas wt (g)	0.18 ± 0.01	0.20 ± 0.01	0.16 ± 0.02
NEFA (mM)			
Day 14	3.20 ± 0.17	3.58 ± 0.07	3.27 ± 0.13
Day 35	3.46 ± 0.42	3.93 ± 0.22	2.82 ± 0.40
Day 70	2.90 ± 0.19	3.44 ± 0.13*	1.92 ± 0.09^+^
Plasma adiponectin (ng/ml)	35.6 ± 7.0	8.2 ± 1.2^+^	0.89 ± 0.13^+^
Plasma TNFα (pg/ml)	34.0 ± 5.7	33.0 ± 2.4	42.3 ± 7.1

* P < 0.05; + P < 0.001; n = 6

Liver weight was increased by CLA and by CLA enriched with either isomer, c9, t11-enriched CLA having the smallest effect. The weight of the pancreas was unaffected by all treatments (Tables 1 and 2). Others have reported that t10, c12- and c9, t11-CLA increase liver weight in lean mice, the effect of the t10, c12 isomer being associated with increased liver lipid [35].

Body length was reduced by CLA (Table 2).

### Glucose tolerance

**Figure 2:**
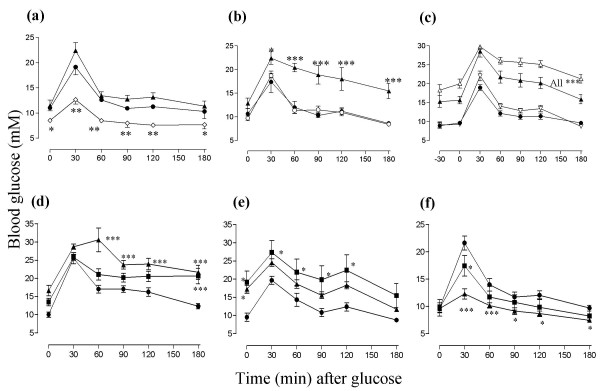
Oral glucose tolerance. Symbols are the same as in Figure 1 and again (a) is experiment 1, (b) is experiment 2 and (c) is experiment 3. (d), (e) and (f) are oral glucose tolerance curve on days 14, 35 and 70 for experiment 4.

**Figure 3:**
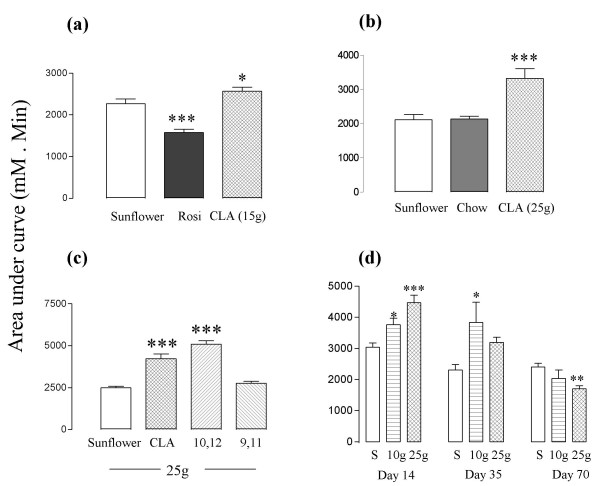
Areas under the glucose tolerance curves shown in Figure 2. Panel (d) shows areas from Figure 2 (d), (e) and (f). Doses of CLA are shown as g/kg diet. *P < 0.05; **P < 0.01: ***P < 0.001 compared to sunflower oil group.

Rosiglitazone lowered fasting blood glucose, improved glucose tolerance (Figure 2a) and reduced the area under the glucose tolerance curve (Figure 3a). Two weeks treatment with CLA or with CLA enriched with t10, c12-CLA raised fasting blood glucose, exacerbated glucose tolerance (Figures 2b to 2d) and increased the area under the glucose tolerance curve (Figures 3b to 3d) compared to the sunflower oil and chow alone diets. CLA-enriched with c9, t11-CLA had no effect (Figures 2c and 3c).

After five weeks of treatment the higher dose of CLA raised glucose levels less than it had after two weeks (Figures 2d, e; 3d, e), and after 10 weeks this dose actually improved glucose tolerance (Figure 2f) and reduced the area under the glucose tolerance curve (Figure 3d). Its effect was similar to that of rosiglitazone over two weeks in experiment 1 (Figures 2a and 3a). The lower dose reduced the peak glucose level after 10 weeks, but it did not reduce the area under the glucose tolerance curve significantly.

### Insulin

**Figure 4:**
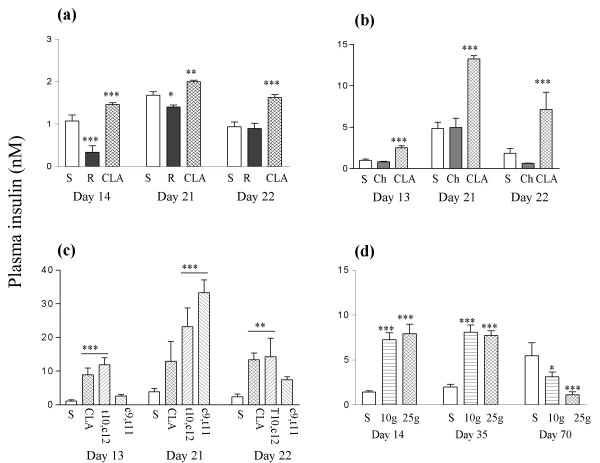
Plasma insulin values from (a) experiment 1, (b) experiment 2, (c) experiment 3, and (d) experiment 4. Doses are described in Methods and the legend to Figure 1. In panel (d) the does of CLA are in g/kg diet. Day 13 or 14, day 35 and day 70 values are following a 4.5 h fast; day 22 values are following a 16 h fast; and day 21 values are for fed animals. Doses of CLA are shown as g/kg diet. *P < 0.05; **P < 0.01: ***P < 0.001 compared to sunflower oil group.

The improvement in glucose tolerance in response to rosiglitazone was associated with a reduction in the fasting plasma insulin level, but rosiglitazone did not reduce the terminal fasting insulin level (Figure 4a). The exacerbations of glucose tolerance in response to two weeks treatment with CLA and CLA enriched with t10, c12-CLA were associated with increases in the fasting plasma insulin level. CLA enriched with c9, t11-CLA did not alter the fasting insulin level on the day that it showed no effect on glucose tolerance. However, c9, t11-enriched CLA increased the plasma insulin level in fed mice on day 21 (Figure 4c).

After five weeks both doses of CLA still raised the fasting plasma insulin level, but after ten weeks, both doses reduced the plasma insulin level compared to the control group (Figure 4d).

### Insulin resistance

**Figure 5:**
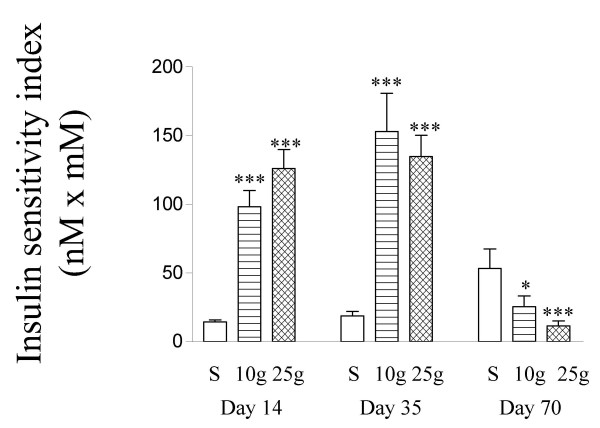
Effect of CLA on insulin sensitivity in experiment 4. The insulin sensitivity index was determined by multiplying the fasting blood glucose concentration (Figure 2d, e, f, 0 min) by the fasting plasma insulin concentration (Figure 4d).

A useful indicator of insulin resistance can be obtained by multiplying values for fasting blood glucose and plasma insulin concentrations [36]. This is analogous to homeostatic model assessment (HOMA), which has been developed for studies in humans, although the full version, which originally involved dividing the product of glucose (mM) and insulin (mU/ml) concentrations by 22.5 is not suitable for studies in rodents [37]. By multiplying fasting blood glucose (mM) by plasma insulin (nM) we illustrate in Figure 5 how CLA initially exacerbated but subsequently improved insulin resistance.

### Triglycerides and fatty acids

**Figure 6:**
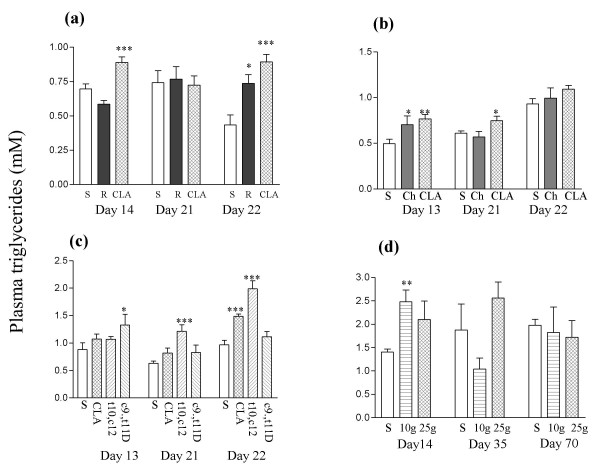
Plasma triglyceride values from (a) experiment 1, (b) experiment 2, (c) experiment 3, and (d) experiment 4. Doses are described in Methods and the legend to Figure 1. In panel (d) the does of CLA are in g/kg diet. Day 13 or 14, day 35 and day 70 values are following a 4.5 h fast; day 22 values are following a 16 h fast; and day 21 values are for fed animals. Doses of CLA are shown as g/kg diet. *P < 0.05; **P < 0.01: ***P < 0.001 compared to sunflower oil group.

CLA raised the fasting triglyceride level in experiments 1 to 3 (Figures 6a to 6c), but in experiment 4 the only statistically significant effect was an increase elicited by the lower dose after two weeks (Figure 6d). CLA enriched with t10, c12-CLA had a similar or greater effect than CLA. Surprisingly on day 13 c9, t11-CLA, but not CLA or t10, c12-CLA caused a significant increase in the plasma triglyceride level, although c9, t11-CLA did not have a significant effect compared to CLA (Figure 6c).

CLA and isomer-enriched CLAs had no effect on the fasting plasma non-esterified fatty acid (NEFA) concentration in experiment 3 (Table 1), and CLA had no effect after two and five weeks in experiment 4 (Table 2). After ten weeks, however, the lower dose of CLA raised the plasma concentration of NEFA, whereas the higher dose lowered it (Table 2).

### TNFα and adiponectin

After ten weeks CLA had no effect on the plasma TNFα concentration. The plasma adiponectin concentration, by contrast, was markedly decreased (Table 2).

### PPAR activation

**Figure 7:**
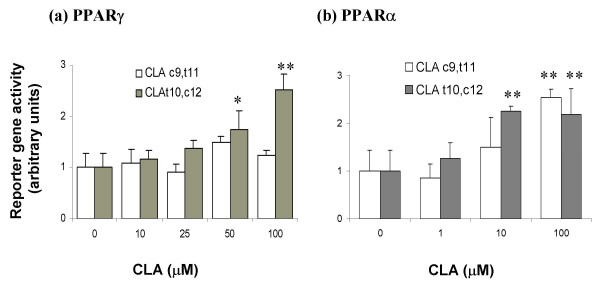
Effect of the c9, t11 and t10, c12-enriched CLA on (**a**) PPARγ- and (**b**) PPARα mediated gene expression. Cos-7 cells were transiently transfected with the plasmids pPPRE3TK-luc, pRLTK, pRSV/hRXRα and pcDNA3/hPPARγ 1 (**a**) or pcDNA3/hPPARα (**b**). Transfected cells were treated for 46 (**a**) or 24 (**b**) hours. Cell extracts were assayed for firefly and renilla luciferase activity. Reporter gene activity was determined by normalising firefly luciferase activity against renilla luciferase activity. The CLA isomers were prepared in 0.1% DMSO so that each of the stated isomers was at the given concentration. * *P *< 0.05 and ** *P *< 0.01; n = 6.

CLA enriched with t10, c12-CLA (50 and 100 μM), but not with c9, t11-CLA, stimulated PPARγ-mediated reporter gene activity (Figure 7a). In contrast, both CLA isomers (100 μM) elicited a significant increase in PPARα-mediated reporter gene expression, and c9, t11-CLA was effective at 10 μM (Figure 7b).

## Discussion

### Physiology

A number of studies have found that CLA reduces weight gain and fat accretion in both rats and mice. Those that have failed to show such effects are generally those that have used low levels of CLA or CLA that contained low concentrations of t10, c12-CLA [6]. Studies disagree, however, as to whether CLA improves or exacerbates glucose tolerance and insulin resistance in these species: studies in rats show improvements [18-21], whereas studies in mice, with the exception of one study in *lep^db^*/*lep^db ^*mice [34], show exacerbations [7-10]. In humans, several studies have shown a loss of fat [1-4], but doubts have been raised about the use of CLA for the treatment of obesity by reports that both t10, c12-CLA and c9, t11-CLA exacerbated insulin resistance in abdominally obese men [11-13], and that t10, c12- (but not c9, t11-CLA) reduced the HDL cholesterol concentration or the HDL:LDL cholesterol ratio [11,12,14]. These are by no means consistent findings, however [13-17].

Our study raises the possibility that an initial exacerbation of glucose tolerance, apparently due to insulin resistance, might, after prolonged treatment with a high dose of CLA, be followed by improved insulin sensitivity and glucose tolerance. We used genetically obese (*lep^ob^*/*lep^ob^*) mice as our model of insulin resistance. There is only one previous report of the effect of CLA in *lep^ob^*/*lep^ob ^*mice [9]. In agreement with other studies in rodents, we found that CLA reduced weight gain and perigenital fat pad weight, and that this effect appeared to be produced by the t10, c12- rather than the c9, t11-isomer. We also found, like other studies in mice, that glucose tolerance was initially exacerbated and the plasma insulin concentration was raised. Again, these effects were primarily due to the t10, c12-isomer. However, in our last experiment we found that after ten weeks glucose tolerance improved and the fasting plasma insulin concentration was reduced by treatment of *lep^ob^*/*lep^ob ^*mice with CLA. These benefits were achieved faster with the higher (25 g/kg diet, including 40% t10, c12-CLA) than the lower dose (10 g/kg diet) of CLA.

Our results are similar to those of Hamura *et al *[34], insofar as they found that treatment of *lep^db^*/*lep^db ^*mice with CLA for eleven weeks improved glucose tolerance and lowered insulin levels during the glucose tolerance test. Hamura *et al*. found that glucose tolerance was also improved in *lep^db^*/*lep^db ^*mice after five weeks of treatment, but this was apparently due to increased insulin secretion rather than improved insulin sensitivity. It is not clear why others have not also found improved glucose tolerance and reduced insulin levels following prolonged treatment of mice with CLA. In the one previous study in *lep^ob^*/*lep^ob ^*mice, the dose of t10, c12-CLA was only about 5.9 g/kg diet and treatment was for only four weeks [9]. A study in lean C57Bl/6J mice similarly used a dose of 4 g/kg diet of t10, c12-CLA for four weeks [10]. Other studies lasted for twelve weeks and eight months, however. The dose of CLA was only 10 g/kg diet in both studies and neither was conducted in exceptionally obese or insulin resistant mice [7,8]. We therefore suggest that improved insulin sensitivity is most likely to be found when mice are initially markedly obese and insulin resistant, and when treatment is prolonged and results in a major loss of adipose tissue. The relevance of our findings to humans is unclear, but it is interesting that in one 12 month study plasma glucose was raised after two weeks treatment with CLA, but not at any subsequent time [16].

After ten weeks the higher dose of CLA lowered the plasma fasting non-esterified fatty acid concentration, which is consistent with improved insulin sensitivity (Table 2). The lower dose raised the NEFA concentration at this time, despite apparently improving insulin sensitivity, even though it did not raise the NEFA concentration at earlier times when glucose tolerance was reduced and plasma insulin levels were raised. This paradox seems to be largely a consequence of the low control NEFA concentration after ten weeks: the highest NEFA level in the low dose CLA group was after five weeks of treatment and it seems possible that the NEFA concentration in the low dose group would have fallen with further treatment, just as it was falling in the high dose group (Table 2). Hamura *et al*. [34] found that after twelve weeks CLA reduced the plasma NEFA concentration in *lep^db^*/*lep^db ^*mice, suggesting that insulin sensitivity was improved. After six weeks, however, CLA raised the NEFA concentration, suggesting that CLA exacerbated insulin sensitivity at this time. The elevated plasma NEFA concentration may have been partly responsible for the elevated plasma insulin and improved glucose tolerance after six weeks in their study. CLA and its major isomers have little or no effect on plasma NEFA levels in humans [11,12,14].

### Mechanism

No single mechanism has been identified that can account for the various effects of CLA on lipid and carbohydrate metabolism, let alone its anticarcinogenic and immunomodulatory activities. In part this is because CLA is a mixture of isomers, each with its own balance of activities. The activities of even the most active isomer, t10, c12-CLA, cannot, however, be pinned down to a single mechanism.

We can first rule out any possibility that responses to CLA in our experiments were mediated by a fall in leptin levels as has been suggested by others [8], because our study was conducted in *lep^ob^*/*lep^ob ^*mice. In any event, when fat loss is achieved by reducing energy intake, insulin action and glucose tolerance improve despite a reduction in the plasma leptin concentration.

Antagonism of PPARγ by t10, c12-CLA has been suggested to contribute to both decreased adipogenesis and insulin sensitivity [38]. A recent report that c9, t11-CLA exacerbates insulin sensitivity [12] is consistent with a report that it too antagonises PPARγ, albeit a little less effectively than t10, c12-CLA [25]. We found little evidence that c9, t11-CLA exacerbates insulin sensitivity in *lep^ob^*/*lep^ob ^*mice, however (Figures 3c and 4c), and others have reported no effect [9].

Some workers favour the hypothesis that t10, c12-CLA decreases adipogenesis and insulin sensitivity by a mechanism that involves decreased expression of PPARγ in adipose tissue [38]. Since PPARγ agonists increase PPARγ expression in some situations [39,40], antagonism of PPARγ might decrease PPARγ expression. By contrast with mice, however, in rats CLA increases both PPARγ mRNA expression and insulin sensitivity [28]. Thus it is possible that CLA activates one mechanism that decreases and another that increases insulin sensitivity, and that PPARγ expression reflects the balance of these forces, rather than having a causal role.

In any event, our results do not support antagonism of PPARγ as a mechanism of action of CLA. In agreement with some other workers [18,33], we find that t10, c12-CLA, but not c9, t11-CLA activates PPARγ. Activation of PPARγ might contribute to the improvement in insulin sensitivity in *lep^ob^*/*lep^ob ^*mice following prolonged treatment with CLA.

Activation of PPARα might also contribute to improved insulin sensitivity. t10, c12-CLA was more potent as an activator of PPARα than of PPARγ in the particular assays that we used, although it is inappropriate to make precise comparisons of potency in view of the different plasmids, their levels and other differences between the two assays. In contrast to some previous reports [10,41], t10, c12-CLA was, if anything, more potent than c9, t11-CLA as an activator of PPARα. Nevertheless, c9, t11-CLA was sufficiently effective to raise the possibility that it contributed to the improvement in insulin sensitivity due to the prolonged treatment with CLA.

Activation of PPARα and peroxisomal proliferation might contribute to the increased liver weight in c9, t11-CLA-treated mice, steatosis also playing a role, at least in the case of t10, c12-CLA [10]. Activation of PPARα does not, however, appear to have a major role in the anti-obesity effect of CLA, because CLA reduced body fat content in PPARα knockout mice [42]. Moreover, some investigators have found that c9, t11-CLA is more effective than t10, c12-CLA as an activator of PPARα [10,41]. Other workers have shown that CLA also activates PPARβ/δ [10,41], but the effect was small in one these studies and c9, t11-CLA was more potent than t10, c12-CLA [10].

We measured plasma TNFα and adiponectin concentrations following prolonged treatment with CLA. Neither hormone contributed to the improved insulin sensitivity at this time: the plasma TNFα concentration was unchanged by CLA, and the plasma adiponectin concentration was reduced rather than increased. Other investigators have reported that t10, c12-CLA but not c9, t11-CLA reduces the level of adiponectin mRNA in white adipose tissue of lean mice [35]. Since adiponectin is released primarily from adipocytes but plasma levels are low in obesity and increased by weight loss [43], the marked reduction in plasma adiponectin in the CLA-treated mice is consistent with their having less, rather than smaller, adipocytes. This is in turn consistent with the view that the anti-obesity effect of CLA and the initial decrease in insulin sensitivity is due to apoptosis of adipocytes. Decreased fat cell number may also account for the tendency of CLA to increase plasma triglyceride levels in lep^ob^/lep^ob ^mice, the remaining fat cells being too full to accommodate the triglyceride released by cells that have undergone apoptosis. Other workers have reported that CLA reduces plasma triglyceride levels; but in some cases this reduction was in lean mice with less replete adipocytes [42,26], and in the one other study in *lep^ob^*/*lep^ob ^*mice, it was an effect of c9, t11-CLA, which presumably causes little apoptosis of adipocytes [9]. Interestingly, CLA has been reported to *increase *plasma adiponectin levels in Zucker diabetic fatty rats, at the same time decreasing plasma triglycerides and improving insulin sensitivity [21]. We therefore suggest that the main effect of CLA in Zucker diabetic fatty rats is to reduce fat cell size, rather than to promote apoptosis and reduce fat cell number.

## Conclusions

Treatment with CLA initially decreased but subsequently increased insulin sensitivity in *lep^ob^*/*lep^ob ^*mice. Activation of both PPARγ and PPARα may contribute to the improvement in insulin sensitivity. In the short term, however, another mechanism, activated by t10, c12-CLA but not c9, t11-CLA, which probably leads to reduced adipocyte number and consequently plasma adiponectin concentration, may decrease insulin sensitivity.

## Methods

### Animals

Female C57Bl/6 *lep^ob^*/*lep^ob ^*mice were obtained from Harlan Olac (Bicester, UK) and maintained at 23 ± 1°C with lights on from 07:00 to 19:00 h. They were housed in plastic cages with bedding and fed 'rat and mouse standard diet' (Beekay Feed, B & K Universal Ltd., Hull, UK). Six days before the start of the studies they were allocated to treatment groups (6 mice per group), such that each group had a similar mean bodyweight. The CLA and other treatments were mixed with powdered diet and given *ad libitum*. The mice were killed by cervical dislocation in experiments 1 to 3; in experiment 4 they were anaesthetised with sodium pentobarbitone (Sagatal; 80 mg/kg, i.p.) and exsanguinated through an aortic catheter. All procedures were conducted in accordance with our Home Office, UK project licence under the Animals (Scientific Procedures) Act and as agreed by the University of Buckingham Ethical Review Board.

### Materials added to diets

CLA (in acid form), including isomer-enriched CLA, and sunflower oil were provided by Loders Croklaan, Wormerveer, The Netherlands. The CLA used in experiments 1, 2 and 3 was Clarinol™ A-60, containing 30% c9, t11-CLA and 31% t10, c12-CLA and 25% oleic acid. The CLA used in experiment 4 was Clarinol™ A-80, containing 40% c9, t11-CLA and 40% t10, c12-CLA. Rosiglitazone was synthesised by Dextra Laboratories, Reading, Berks, UK.

### Experiment 1

At the start of treatment the mice weighed 34.5 ± 4.3 g (mean ± S.D.). The treatment groups were high oleic (83.5%) sunflower oil (control; 15 g/kg diet); rosiglitazone (10 mg/kg diet) plus high oleic sunflower oil (15 g/kg diet); CLA (Clarinol™ A-60: 15 g/kg diet). An oral glucose tolerance test was conducted after 14 days as described below. 30 min prior to giving glucose, when the mice had been fasted for 4.5 hours, blood (100 μl) was taken for the measurement of plasma insulin and triglycerides. Plasma insulin and triglycerides were also measured after 21 days when the mice were feeding *ad libitum*, and after 22 days when they had been fasted for 16 hours, immediately before termination of the experiment.

### Experiment 2

At the start of the treatment the mice weighed 44.0 ± 3.2 g (mean ± S.D.). A group fed on powdered chow alone was included. The doses of CLA (Clarinol™ A-60) and sunflower oil in the other two groups were increased to 25 g/kg diet. The oral glucose tolerance test and other measurements were carried out as described for experiment 1, except that the glucose tolerance test was one day earlier (i.e. after 13 days).

### Experiment 3

At the start of treatment the mice weighed 34.1 ± 2.1 g (mean ± S.D.). The treatment groups were high oleic sunflower oil (25 g/kg diet); CLA (Clarinol ™ A-60: 25 g/kg diet); CLA with the CLA component of Clarinol enriched to 90% with t10, c12-CLA (25 g/kg diet); similarly, CLA enriched to 90% with c9, t11-CLA (25 g/kg diet). The oral glucose tolerance test and other measurements were carried out as described for experiment 2. In addition, the plasma non-esterified fatty acid concentration was measured in the blood taken prior to the glucose tolerance test, and the liver, pancreas, parametrial white adipose tissue depot and interscapular brown adipose tissue depot were weighed at termination.

### Experiment 4

At the start of treatment the mice weighed 31.6 ± 3.8 g (mean ± S.D.). The treatment groups were high oleate (as glyceride) sunflower oil (25 g/kg diet); CLA (Clarinol A80: 10 g/kg diet) plus high oleic sunflower oil (15g/kg diet); CLA (Clarinol A80: 25 g/kg diet). Oral glucose tolerance tests preceded by blood sampling for the measurement of triglycerides, insulin and non-esterified fatty acids were taken after 14, 35 and 70 days of treatment. After 77 days of treatment the animals were anaesthetised in the fed state and blood was taken from the thoracic aorta for the measurement of plasma TNFα and adiponectin. Tissues were weighed as in experiment 3. Body length was measured from the tip of the nose to the anus.

### Oral glucose tolerance tests

In each experiment oral glucose tolerance was measured after 13 or 14 days. In experiment 4 it was also measured after five and ten weeks. The mice were fasted for five hours before being dosed with glucose (3 g/kg, p.o. in experiments 1 and 2; 2 g/kg, p.o. in experiments 3 and 4). Blood samples (20 μl) were taken from the tip of the tail after applying a local anaesthetic (lignocaine) and immediately before and 30, 60, 90, 120 and 180 min after dosing the glucose. They were mixed with haemolysis reagent and blood glucose was measured in duplicate using the Sigma Enzymatic (Trinder) colorimetric method and a SpectraMax 250 (Molecular Devices Corporation, Sunnyvale, CA, USA). Areas under the glucose tolerance curve (0–180 min) and other manipulations and analysis of the data were carried out using Prism software, version 3.0 (GraphPad Software Inc., San Diego, CA, USA).

### Other plasma analytes

Blood was collected into EDTA-coated microcuvettes (Sarstedt microcuvette, Aktiengsellschaft & Co., Nämbrecht, Germany) for the measurement of plasma insulin, non-esterified fatty acids and triglycerides. Plasma was stored at -80°C. 5 μl plasma samples were assayed using kits for triglyceride (Sigma enzymatic colorimetric method), non-esterified fatty acids (Wako Chemicals, Neuss, Germany) and insulin (mouse standard; Crystal Chemistry Incorporated, Downers Grove, IL, USA).

For the measurement of plasma TNFα (Linco; St Charles, MI, USA) and adiponectin (Biosource UK, Nivelles, Belgium), blood was taken into tubes containing 200 units of heparin and plasma was stored at -80°C.

### Plasmids for reporter gene assays

pRSV/hRXRα and pcDNA3/hPPARγ1 were both obtained from Professor VKK Chatterjee (Addenbrooke's Hospital, Cambridge, UK). PPPRE3TK-luc was prepared by replacing the NF-kB enhancer element of pNF-κB-luc (Clontech) with a cassette of 3 PPAR Response Elements (PPREs). A double-stranded PPRE cassette was prepared using the 'Klenow fill-in' technique. An 113 bp oligonucleotide (PPRE3) 5'- GCATTCACGCGTCAAATATAGGCCATAGGTCATTCTCGAGCAAATATAGGCCATAGGTCATTCTCGAGCAAATATAGGCCATAGGTCAGATTCGATCAATATAGGCCATAGGTCACTCGAGGCAACAGATCTTACGCATG -3' containing a triplet of PPREs and appropriate restriction endonuclease sites was used as a template for synthesis of a second DNA strand. This was primed by PPRE3R, 5'-CATGCGTAAGATCTGTTGCC-3', which is complementary to the 3' region of PPRE3. 20 μl annealed PPRE3 and PPRE3R, 1.5 μl 2 mM dNTPs, 1 × Klenow Buffer and 5 units DNA Polymerase I (Klenow fragment), were incubated for 1 hour at 37°C and then 10 minutes at 75°C, purified by ethanol precipitation and resuspended in sterile water. The double-stranded PPRE cassette was digested with *Mlu*I and *Bgl*II and ligated into pNF-κ B-luc that had been cleaved with the same restriction endonucleases. Ligated DNA was transformed into competent JM109, *E. coli *cells (Promega). pcDNA3/PPARα was prepared by removing the human PPARα cDNA insert from pUC18/hPPARα as a *Nru*I/*Bam*HI fragment and ligating it into *Eco*RV/*Bam*HI cleaved pcDNA3.1(-) (Invitrogen). Ligated DNA was transformed into competent JM109, *E. coli *cells (Promega).

### PPARγ and PPARα reporter gene assays

Cos-7 cells (ECACC No. 87021302) were routinely cultured in DMEM containing 10% FCS, 2 mM L-Glutamine, 100 iu/ml penicillin and 100 μg/ml streptomycin at 37°C/5% CO_2_. Transient transfections were performed using LipofectAMINE as directed by the manufacturers (GibcoBRL). For the PPARγ assay Cos-7 cells were plated in 24-well plates at a density of 0.375 × 10^5 ^cells/well. Cells were transfected in serum-free medium (DMEM containing 2 mM L-glutamine) with pPPRE3TK-luc, pRLTK, pRSV/hRXRα and pcDNA3/hPPARγ 1 at concentrations of 0.4, 0.03, 0.02 and 0.02 μg/well, respectively. Five hours after transfection cells were fed with 250 μl/well of serum-free medium containing various concentrations (0–100 μM) of CLA (either the c9, t11 isomer or t10, c12 isomer prepared in 0.1% DMSO). Cell lysates were prepared after 46 hours using 100 μl 1 × passive lysis buffer (Promega) per well. Firefly and renilla luciferase activities were measured using a Dual Luciferase Assay kit (Promega), as described by the manufacturers. Measurements were performed on an MLX microtitre plate luminometer (Dynex). For the PPARα assay Cos-7 cells were plated in 24-well plates at a density of 0.5 × 10^5 ^cells/well. Cells were transfected in serum-free medium with pPPRE3TK-luc, pRLTK, pRSV/hRXRα and pcDNA3/hPPARα at concentrations of 0.4, 0.04, 0.03 and 0.03 μg/well, respectively. Five hours after transfection DMEM supplemented with 2 mM L-glutamine and 20% SBCS (charcoal-stripped bovine calf serum, Sigma) was added to the cells. Following 18 hours incubation at 37°C/5% CO_2 _the medium was removed and replaced with medium (DMEM supplemented with 2 mM L-glutamine and 10% SBCS) containing various concentrations (0-100 μM) of CLA enriched to 85% with the c9, t11 isomer or 81% with the t10, c12 isomer prepared in 0.1% DMSO. After 24 hours of treatment cell lysates were prepared and luciferase activity measured as described above.

### Statistics

Data were analysed by one-way analysis of variance followed by LSD test with the sunflower oil treatment as the control. Means are of 6 values with SEM.

## Authors' contributions

MC, LB, IM and MS devised the experiments. EW, MS and CS, supported by JO and SW conducted the *in vivo *experiments and analysed materials from these. AM conducted the *in vitro *experiments. MS and MC conducted the initial analysis and interpretation of the data. JA reanalysed some of the data and wrote the manuscript with input from the other authors, and in particular from AE and IM with respect to interpretation and perspectives.
